# Direct comparison of methionine restriction with leucine restriction on the metabolic health of C57BL/6J mice

**DOI:** 10.1038/s41598-017-10381-3

**Published:** 2017-08-30

**Authors:** Emma K. Lees, Ruth Banks, Chelsea Cook, Sophie Hill, Nicola Morrice, Louise Grant, Nimesh Mody, Mirela Delibegovic

**Affiliations:** 1 Institute of Medical Sciences, School of Medicine, Medical Sciences and Nutrition, University of Aberdeen, Aberdeen, UK; 2 School of Health Sciences, Liverpool Hope University, Liverpool, UK

## Abstract

The effects of methionine restriction (MR) in rodents are well established; it leads to decreased body and fat mass, improved glucose homeostasis and extended lifespan, despite increased energy intake. Leucine restriction (LR) replicates some, but not all, of these effects of MR. To determine any differences in metabolic effects between MR and LR, this study compared 8 weeks of MR (80% restriction), LR (80% restriction) and control diet in 10-month-old C57BL/6J male mice. Body composition, food intake and glucose homeostasis were measured throughout the study and biochemical analyses of white adipose tissue (WAT) and liver were performed. MR and LR decreased body and fat mass, increased food intake, elevated lipid cycling in WAT and improved whole-body glucose metabolism and hepatic insulin sensitivity in comparison to the control diet. MR produced more substantial effects than LR on body mass and glucose homeostasis and reduced hepatic lipogenic gene expression, which was absent with the LR diet. This could be a result of amino acid-specific pathways in the liver responsible for FGF21 stimulation (causing varied levels of FGF21 induction) and Akt activation. In summary, LR is effective at improving metabolic health; however, MR produces stronger effects, suggesting they activate distinct signalling pathways.

## Introduction

Methionine restriction (MR) is a dietary technique, which consistently reduces body weight and adiposity levels and prevents high-fat diet (HFD) -induced obesity, despite increasing food consumption, in rodents^1–3^. MR is capable of producing these effects due to its ability to enhance energy expenditure (EE), through activation of uncoupling protein 1 (UCP1) -dependent and UCP1-independent non-shivering thermogenesis in white adipose tissue (WAT) and brown adipose tissue (BAT), which elevates energy demands^1^.

Browning of WAT can occur after prolonged adrenergic stimulation and leads to brown-like adipocytes forming in WAT, increased oxidative capacity and elevated expression of UCP1^4^. There is evidence for MR dietary treatment increasing β-adrenergic stimulation and leading to browning of WAT and remodelling of lipid metabolism, but also enhancing lipid cycling in WAT^5–8^. Lipid cycling is the process of glucose being used for *de novo* lipogenesis prior to lipids being oxidised and wastes more potential energy as heat (that could be used for ATP synthesis), versus directly oxidising glucose^9–12^. This process of lipid cycling by dietary MR, together with its ability to increase UCP1 activity could explain the increased energy expenditure^1^.

In the liver, MR decreases lipogenesis and increases free fatty acid (FFA) oxidation^5, 6, 8^. Together with the diet’s effect to reduce triglyceride (TG) storage and decrease TG levels, it protects against fatty liver disease^3, 5^. Furthermore, MR extends lifespan in rats and mice^3, 13, 14^, decreases inflammation^15^ and improves whole-body glucose homeostasis and insulin sensitivity^16–19^. MR has been shown to have methionine-specific effects on hepatic insulin sensitivity by causing lower production of cysteine which is an essential precursor of glutathione (GSH)^17^. GSH activates the phosphatase; phosphatase and tensin homologue (PTEN), which degrades phosphatidylinositol (3,4,5)-trisphosphate (PIP3), leading to lower activation of Akt^17^. Therefore, with lower GSH being produced with the MR diet, PTEN is less active, degradation of PIP3 is reduced and downstream stimulation of Akt is enhanced^17^.

Other essential amino acids (EAAs), apart from methionine, have been examined for their metabolic effects. In mice, tryptophan restriction increases lifespan^20^ and total amino acid restriction also extends lifespan^21^. Complete deprivation of leucine for 7 days results in body weight loss and decreased adiposity, through increased energy expenditure, via increased sympathetic nervous system activity and UCP1 expression in BAT^22, 23^. Leucine deprivation improves hepatic insulin sensitivity through activating general control nonderepressible 2 (GCN2), which reduces the inhibition of insulin receptor substrate 1 (IRS1) by the mammalian target of rapamycin (mTOR); therefore, enhancing downstream insulin signalling^24^. By activating GCN2, leucine deprivation also leads to induction of the hormone fibroblast growth factor 21 (FGF21) in the liver^25^. GCN2 senses the presence of uncharged transfer RNA (tRNA) and responds by phosphorylating eukaryotic initiation factor 2α (eIF2α), causing reduced global protein synthesis but increased transcription of activating transcription factor 4 (ATF4)^25^. ATF4 activation leads to increased transcription of FGF21 and therefore, release from the liver^25^. FGF21 is essential for the effects of leucine deprivation on lipid metabolism^26^. There is also substantial evidence for MR to increase FGF21 release from the liver^17, 27, 28^ and for the effect of MR on energy expenditure to be dependent on FGF21^27^.

FGF21 is a hormone, which regulates growth and normally is released in response to starvation by a peroxisome proliferator-activated receptor (PPAR) α mechanism^29, 30^. FGF21 induces browning of WAT, shown through increased expression of oxidative genes^31^, by signalling through the FGF receptor (FGFR) and co-receptor, β Klotho^32^. FGF21 treatment of HFD-fed mice improved glucose homeostasis and increased energy expenditure, leading to significant loss of body and fat mass^33^. In the liver, FGF21 decreases lipid accumulation through lowering lipogenesis^33–36^ and enhancing fatty acid oxidation^37^.

Leucine restriction (LR) of 85% was recently examined compared to a control diet and was found to reduce body weight and accumulation of adipose tissue, increase food intake, improve glucose tolerance and stimulate hepatic induction of FGF21^38^. However, 85% LR did not have any effect on hepatic lipogenesis, unlike MR’s ability to decrease lipogenic gene expression in the liver, but it did lead to remodelling of adipose tissue^38^. This study suggests that LR produces many of the same metabolic effects as MR, but there are some differences between the effects produced by the diets^38^. It also remains to be determined whether the diets produce the same magnitude of effect for those effects that they have in common.

A direct comparison of methionine and leucine restriction has not previously been examined; therefore, this study aimed to compare 80% MR with 80% LR in order to identify the similarities and differences in metabolic effects and size of these effects between these two diets.

## Results

### MR and LR decrease body mass and alter body composition

**Figure 1:**
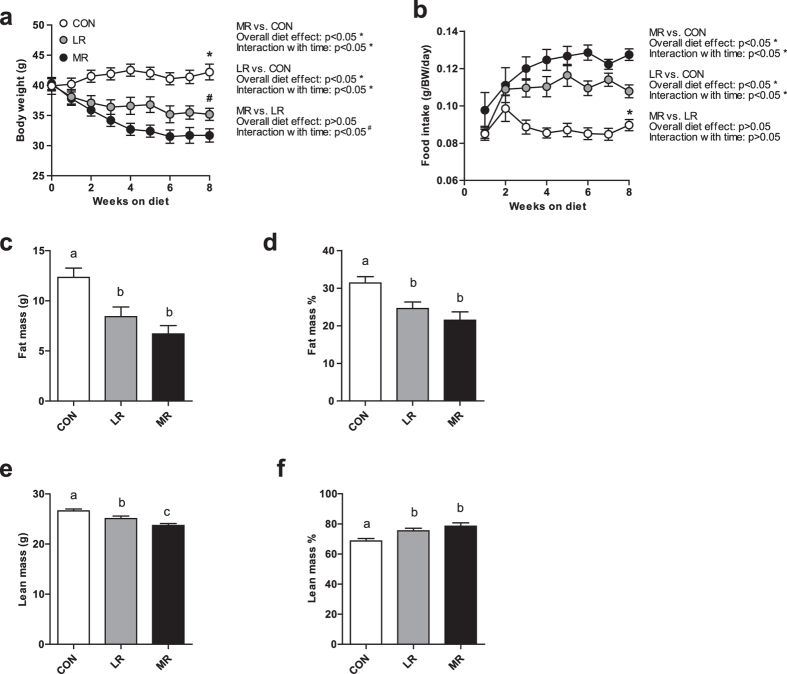
Body mass, food intake, fat mass and lean mass of mice on MR (*n* = 10), LR (*n* = 10) and control (*n* = 9) diet. (**a**) Body mass (**g**) and (**b**) food intake (g/BM/day) measurements from 8 weeks of dietary treatment. Significance was calculated by repeated measures two-way ANOVA. *Significantly different to MR and LR-fed mice (*P* < 0.05) for overall diet effect and interaction of diet with time. ^#^Significantly different to MR-fed mice (*P* < 0.05) for interaction of diet with time. (**c**) Fat mass (**g**), (**d**) fat mass (%), (**e**) lean mass (g), (**f**) lean mass (%) were measured after 3 weeks on diet by MRI scan in mice fed MR, LR or control diet. Significance was calculated by two-tailed Student’s t-test. ^a,b^Means with letters that differ denote significance at level of *P* < 0.05. Data are represented as mean ± SEM. White circles/bars, control-fed mice; grey circles/bars, LR-fed mice; black circles/bars, MR-fed mice.

Control-fed mice maintained a stable body mass throughout the course of the study, which only increased slightly by 2.3 g (Fig. 1a). However, MR- and LR-fed mice both significantly decreased their body mass over the course of the study, compared to control-fed mice (Fig. 1a). MR-fed mice decreased body mass by 8.6 g and LR-fed mice reduced body mass by 4.9 g during the 8 week treatment, the overall effect of the diets was not significant; however, MR and LR produced a significantly different effect on body mass over time (Fig. 1a). After approximately 3 weeks on diet, MR produces a greater decline in body mass than LR (Fig. 1a). MR and LR diet-fed mice had significantly higher levels of food intake (adjusted for body mass) during the course of the study compared to control-fed mice, whereas there were no measurable differences between LR and MR (Fig. 1b). Both MR and LR significantly decreased levels of fat mass compared to control diet in terms of both total amount (g) and as a proportion of body mass (%) (Fig. 1c,d). LR and MR diets did not differ for levels of fat mass (Fig. 1c,d). Similarly, the two dietary treatments also significantly decreased total amount of lean mass (g) compared to control diet and MR significantly reduced lean mass (g) compared to LR diet; however, this was a result of losing both, fat and lean mass, but predominantly fat mass (Fig. 1e). This is shown in the finding of lean mass as a proportion of body mass (%) being significantly higher in both MR and LR diets than control diet (Fig. 1f). MR and LR diet did not produce a significantly different effect on proportion of lean mass (%) (Fig. 1f).

### MR and LR improve whole-body glucose metabolism

**Figure 2:**
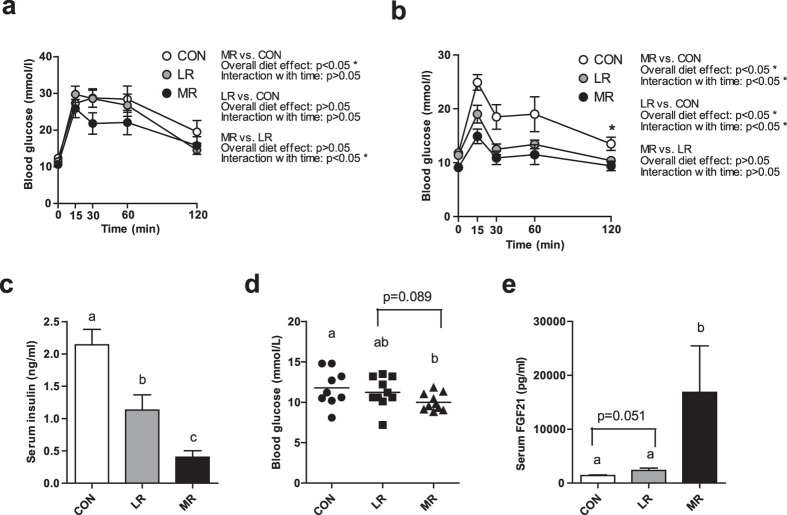
Whole-body glucose homeostasis and serum FGF21 levels of mice on MR, LR or control diet. Glucose tolerance was assessed after (**a**) 2 days on diet (data presented previously for MR vs. CON^28^) and (**b**) 5 weeks on diet by a GTT (2 g/kg) after a 5 h fast in mice fed MR (*n* = 10), LR (*n* = 10) or control (*n* = 9) diet. Significance was calculated by repeated measures two-way ANOVA. *Significantly different to MR and LR-fed mice (*P* < 0.05) for overall diet effect and interaction of diet with time. (**c**) Fasting serum insulin was measured after 6 weeks on diet, (**d**) fasting blood glucose was measured after 8 weeks on diet, (**e**) fasting serum FGF21 was measured after 6 weeks on diet, using tail vein blood samples taken after a 5 h fast in mice fed MR (*n* = 6–10), LR (*n* = 8–10) or control (*n* = 9) diet. Significance was calculated by two-tailed Student’s t-test. ^a,b,c^Means with letters that differ denote significance at level of *P* < 0.05. Data are represented as mean ± SEM. White circles/bars, control-fed mice; grey circles/bars, LR-fed mice; black circles/bars, MR-fed mice.

We examined markers of whole-body glucose homeostasis by performing glucose tolerance tests (GTT) after 2 days and 5 weeks on diet, by measuring fasting serum insulin levels after 6 weeks on diet and by analysing fasting blood glucose at the end of the study. After just 2 days on diet MR significantly improved glucose tolerance compared to control diet (data published previously^28^) (Fig. 2a). In comparison of MR and LR, the overall effect of the diet was not significant; however, they produced a significantly different effect on glucose tolerance over the 2-hour GTT (Fig. 2a). LR was unable to improve glucose maintenance at this time point in comparison to control diet (Fig. 2a). These improvements by MR occurred before any changes in body weight/adiposity were apparent.

MR and LR both significantly improved systemic glucose clearance relative to control diet at 5 weeks of dietary regimen (Fig. 2b). There was no significant difference between MR- and LR-fed mice for glucose tolerance at this time point (Fig. 2b). By week 6 of dietary treatment MR and LR significantly decreased fasting serum insulin levels relative to control diet, and MR produced significantly lower fasting serum insulin levels compared to LR diet (Fig. 2c). By the end of the study MR had significantly lowered fasting blood glucose levels compared to control diet and trended towards lower levels than LR diet (p = 0.089), whilst LR had no effect compared to control diet (Fig. 2d).

We also measured fasting FGF21 levels after 6 weeks on diet. MR-fed mice had significantly elevated fasting serum levels of FGF21 compared to LR-fed and control-fed mice (Fig. 2e). LR diet increased fasting serum levels of FGF21 relative to control diet, although this did not reach significance (p = 0.051) (Fig. 2e). Fasting serum IGF-1 levels were measured and no differences were found between any of the groups (data not shown).

### MR and LR increase lipogenic gene expression in epididymal WAT

In order to assess the extent of MR and LR effects on glucose and lipid homeostasis we used qPCR to evaluate changes in gene expression in epididymal WAT and liver of control-, LR- and MR-fed mice.

**Figure 3:**
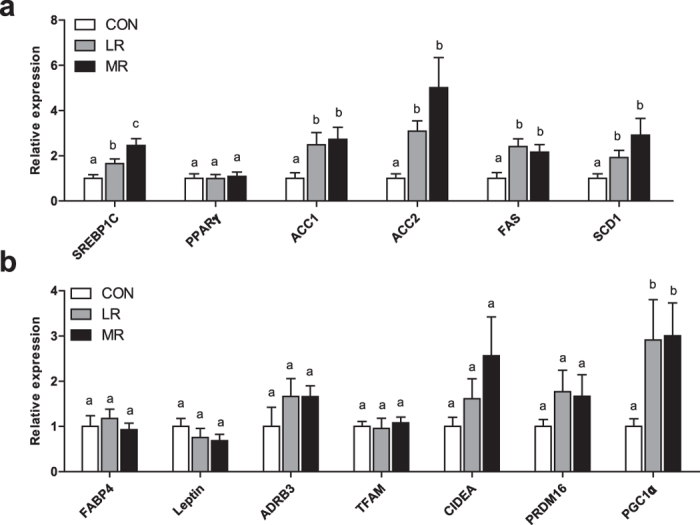
Epididymal WAT gene expression in mice on MR (*n* = 9–10), LR (*n* = 8–10) and control (*n* = 7–9) diet. qPCR was used to measure gene expression in epididymal WAT of (**a**) lipogenic and adipogenic genes and (**b**) thermogenic and oxidative genes in mice fed MR, LR or control diet. Data were analysed as fold change relative to control-fed mice. Significance was calculated by two-tailed Student’s t-test. ^a,b,c^Means with letters that differ denote significance at level of *P* < 0.05. Data are represented as mean ± SEM. White bars, control-fed mice; grey bars, LR-fed mice; black bars, MR-fed mice.

MR and LR significantly increased the expression of genes which promote lipogenesis; *Sterol regulatory element binding protein 1c* *(Srebp1c*), *Acetyl CoA carboxylase* (*Acc*) *1*, *Acc2*, *Fatty acid synthase* (*Fas*) and S*tearoyl-CoA desaturase 1* (*Scd1*) in WAT compared to control diet (Fig. 3a). There were no differences between MR and LR in expression of these genes in WAT, except for *Srebp1c*, which was significantly higher in MR than LR (Fig. 3a). There were no differences between any of the diets in expression levels of *Pparγ*, *Fatty acid binding protein 4* (*Fabp4*), *Leptin*, β_3_-*adrenergic receptor* (*Adrb3*), *Mitochondrial transcription factor A* (*Tfam*), *Cidea*, and *PR domain containing 16* (*Prdm16*) in WAT (Fig. 3a,b). Expression of *PPAR gamma coactivator 1 alpha* (*Pgc1α*) was increased by both MR and LR diet in WAT, but did not differ between MR and LR diet (Fig. 3a).

### MR decreases lipogenic gene expression in liver

**Figure 4:**
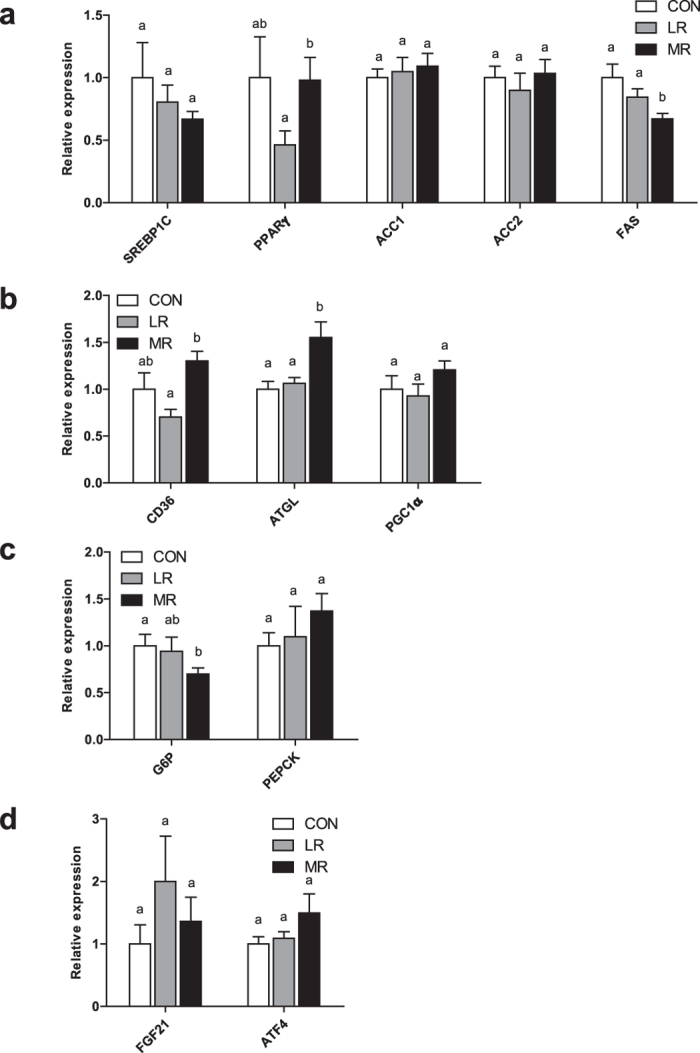
Hepatic gene expression in mice on MR (*n* = 9–10), LR (*n* = 8–10) and control (*n* = 7–9) diet. qPCR was used to measure gene expression in the liver of (**a**) lipogenic genes (**b**) lipolytic and oxidative genes (**c**) gluconeogenic genes and (**d**) *FGF21* and *ATF4* in mice fed MR, LR or control diet. Data were analysed as fold change relative to control-fed mice. Significance was calculated by two-tailed Student’s t-test. ^a,b^Means with letters that differ denote significance at level of *P* < 0.05. Data are represented as mean ± SEM. White bars, control-fed mice; grey bars, LR-fed mice; black bars, MR-fed mice.

In the liver, LR decreased lipogenic gene expression of *Pparγ* compared to MR diet, but expression levels did not differ to control diet (Fig. 4a). MR diet showed no difference to control diet for hepatic gene expression of *Pparγ* (Fig. 4a). MR significantly lowered expression of *Fas* compared to both diets, but there were no differences between control and LR diet (Fig. 4a). There were no changes between any of the diets for expression of *Srebp1c, Acc1*, and *Acc2* in the liver (Fig. 4a). None of the diets showed any differences for expression of *Pgc1α*, but MR significantly increased expression of *Adipose triglyceride lipase* (*Atgl*) compared to both diets and increased expression of *Cluster of differentiation 36* (*Cd36*) compared to LR diet (Fig. 4b). LR and control diet did not differ for either of these two genes and MR and control diet showed no differences in expression of *Cd36* (Fig. 4b). There were no differences between any of the diets for *Phosphoenolypyruvate carboxykinase* (*Pepck*); however, *Glucose 6-phosphate* (*G6p*) expression was significantly lower in MR diet than control diet (Fig. 4c). LR diet showed no differences in *G6p* to either control or MR diet (Fig. 4c). None of the diets showed any differences for mRNA expression of *FGF21 or Activating transcription factor 4* (*Atf4*) (Fig. 4d).

### MR and LR effects on insulin signalling in epididymal WAT and liver

**Figure 5:**
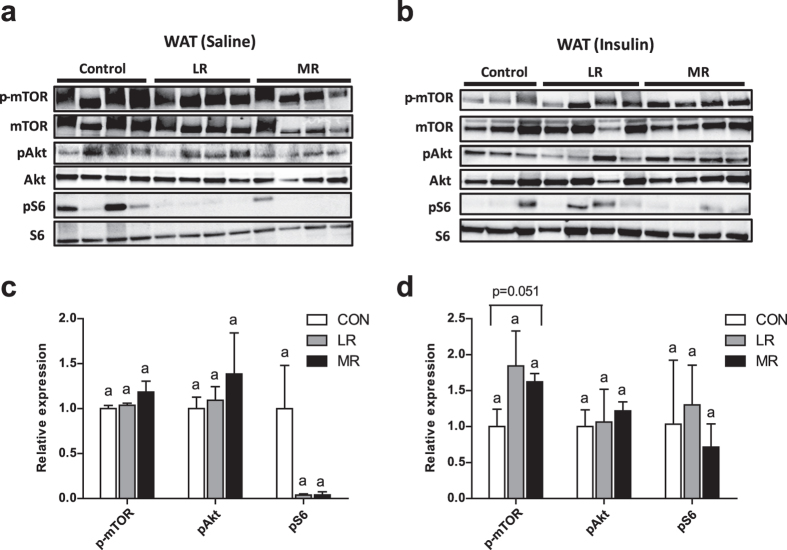
Epididymal WAT insulin signalling in mice on MR (*n* = 4), LR (*n* = 4) and control (*n* = 3–4) diet. Insulin signalling was assessed by administering either (**a**) saline (154 mmol/l NaCl) or (**b**) high dose of insulin (10 mU/g body weight) via i.p. injection after a 5 h fast. Levels of phosphorylated and total mTOR (s2448), Akt (s473) and S6 (s235/236) were measured my immunoblotting. Immunoblots were normalised to total protein for (**c**) saline condition and (**d**) insulin-stimulated condition. Data were analysed as fold change relative to control-fed mice. Significance was calculated by two-tailed Student’s t-test. ^a,b^Means with letters that differ denote significance at level of *P* < 0.05. Data are represented as mean ± SEM. White bars, control-fed mice; grey bars, LR-fed mice; black bars, MR-fed mice.

To assess insulin-dependent signalling, mice were injected with saline (154 mmol/l NaCl) or a high dose of insulin (10 mU/g body weight ﻿for 10 minutes) after a 5-h fast. Firstly, epdidymal WAT was examined. In epididymal WAT, there were no differences between any of the diets for protein expression of components of the insulin signalling pathway, in response to fasting (Fig. 5a,c). Similar results were found in response to insulin stimulation; however, MR did increase levels of phosphorylation of mTOR compared to control diet, but this did not reach significance (p = 0.051) (Fig. 5b,d). MR and LR did not differ for phosphorylation of mTOR (Fig. 5b,d). No differences were found between any of the diets for levels of phosphorylation of Akt or S6 in response to insulin stimulation (Fig. 5b,d).

**Figure 6:**
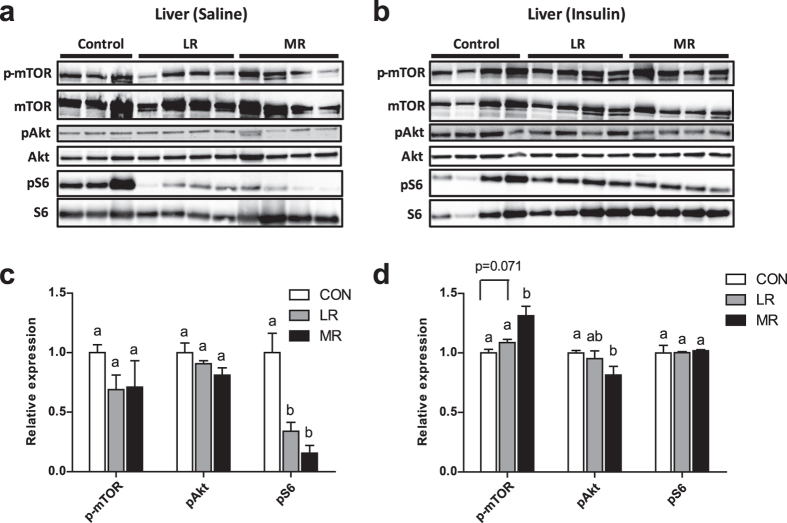
Hepatic insulin signalling in mice on MR (*n* = 4), LR (*n* = 4) and control (*n* = 3–4) diet. Insulin signalling was assessed by administering either (**a**) saline (154 mmol/l NaCl) or (**b**) high dose of insulin (10 mU/g body weight) via i.p. injection after a 5 h fast. Levels of phosphorylated and total mTOR (s2448), Akt (s473) and S6 (s235/236) were measured my immunoblotting. Immunoblots were normalised to total protein for (**c**) saline condition and (**d**) insulin-stimulated condition. Data were analysed as fold change relative to control-fed mice. Significance was calculated by two-tailed Student’s t-test. ^a,b^Means with letters that differ denote significance at level of *P* < 0.05. Data are represented as mean ± SEM. White bars, control-fed mice; grey bars, LR-fed mice; black bars, MR-fed mice.

Next, in exactly the same way as epididymal WAT, hepatic insulin signalling was examined. In the liver, both MR and LR significantly lowered phosphorylation of S6 under fasting conditions (Fig. 6a,c). MR and LR did not differ for levels of S6 phosphorylation in the liver after saline injection (Fig. 6a,c). There were no differences between diets for levels of phosphorylation of mTOR or Akt under fasting conditions (Fig. 6a,c). In response to insulin stimulation, MR-fed mice had significantly higher levels of phosphorylation of mTOR compared to both control and LR-fed mice and LR-fed mice trended towards higher levels of phosphorylation of mTOR relative to control diet (p = 0.071) (Fig. 6b,d). MR significantly decreased levels of Akt phosphorylation when exposed to a high dose of insulin; whereas, LR did not differ to control or MR diet (Fig. 6b,d). None of the diets differed for insulin-stimulated levels of phosphorylation of S6 (Fig. 6b,d).

## Discussion

Dietary MR treatment decreased body mass and adiposity and increased food intake and glucose homeostasis in 10-month old male C7BL/6 J mice compared to control diet. These beneficial metabolic effects are consistent with previous studies investigating the effects of MR in young and adult rodents^3, 16, 17, 19^. LR also produced significantly lower body mass and fat mass and enhanced glucose homeostasis compared to the control diet. In LR-fed mice, it appears that food intake has been maintained at a normal level; therefore, the reduction in body mass has not been compensated for with a reduction in food intake. In some aspects of metabolic health that we assessed, MR showed a greater enhancement than LR, in particular for the reduction in body mass and lowering of serum insulin and blood glucose levels.

Leucine deprivation for 7 days rapidly decreased body weight and fat mass of C57BL/6 mice, probably due to its ability to increase energy expenditure, through elevation of thermogenic, lipolytic and oxidative genes in WAT^22^. Leucine deprivation also lowers blood glucose and serum insulin levels as well as improving hepatic insulin sensitivity^24^. Complete deprivation of leucine in the diet of mice demonstrates very similar effects on whole-body metabolism to LR in this study. Leucine deprivation however, is not a realistic long-term intervention, as there is an increased risk of mortality with EAA deficiency, if treatment is prolonged for more than 3 weeks^8^.

Fasting serum levels of FGF21 were significantly elevated by MR in comparison to both LR and control diet. LR also increased fasting serum FGF21 levels compared to control diet. This corresponds with previous studies showing leucine deprivation stimulates hepatic release of FGF21 through increased activation of GCN2^25^.

To determine if the same pathway is responsible for MR to increase FGF21, a study using GCN2^−/−^ mice delineated that MR was still able to fully phosphorylate eIF2α, increase ATF4, stimulate FGF21 release and produce its metabolic effects independently of GCN2^27^. This was done through protein kinase R-like endoplasmic reticulum kinase (PERK) activation, in response to low GSH levels, rather than its commonly known stimulator of endoplasmic reticulum stress^27^. PERK also activates nuclear factor (erythroid-derived 2)-like 2 (NRF2) to produce the antioxidant response program^39^, which responds to low GSH levels^40^. This mechanism of MR to produce its metabolic effects through lowering GSH was shown in a study, which added cysteine to the MR diet^27^. This increased GSH and lowered serum FGF21, reversed the effects of MR on energy expenditure and serum insulin, as well as blocked MR’s ability to activate PERK and eIF2α and increase NRF2-sensitive and ATF4-sensitive genes in the liver^27^. Therefore, this potential divergence in FGF21-induction pathway could be the cause of the variation in levels of FGF21 in the serum between MR and LR.

As shown before, MR increased lipogenic gene expression in WAT, including *Srebp1c*, *Acc1*, *Acc2*, *Fas* and *Scd1* compared to the control diet^5, 6, 8^. LR elevated the expression levels of the same genes in epididymal WAT, relative to the control diet, producing similar effects to MR. MR and LR had little effect on oxidative and ‘browning’ genes in epididymal WAT, except for both diets increasing *Pgc1α* compared to control diet. This was surprising, as MR has previously been shown to increase sympathetic stimulation of adipose tissue^1^; however, there was a large amount of variability between mice in the current study. The increase in *Pgc1α* is consistent with previous findings of MR elevating oxidative gene expression, which, in combination with the increased lipogenesis, leads to lipid cycling, creating metabolic inefficiency and increased energy expenditure^1, 5–8^.

In the liver, compared to control and LR diet, MR decreased lipogenic (*Fas*) but enhanced lipolytic (*Atgl*) gene expression, consistent with previous studies on MR, where the diet decreased hepatic lipogenesis, increased FA oxidation^5, 6, 8^ and lowered levels of stored hepatic TGs^3, 5^. LR, however, had no measurable effect compared to control diet on hepatic lipogenesis and lipolysis, consistent with previous findings of 80% LR, which concluded that the effects of MR on hepatic lipid metabolism may be exclusive to methionine^38^. The concept of methionine-specific effects in the liver is in line with a previous study, which found an improvement in hepatic insulin sensitivity that was unique to methionine, through its effect of lowering cysteine levels^17^.

FGF21 treatment of diet-induced obese mice resulted in lower body weight and fat mass^33^. A recent study using FGF21^−/−^ mice demonstrated that MR’s ability to increase energy expenditure is dependent on FGF21^27^. This dependency on FGF21 to increase energy expenditure^27^ and the larger rise in serum FGF21 levels with MR versus LR, could explain the more extensive effects on body mass by MR than LR. In addition, this could offer an explanation for the greater effects of MR than LR on glucose homeostasis, as FGF21 administered to diet-induced obese mice lowered serum insulin and blood glucose levels^35^ and in another study, improved whole-body glucose homeostasis independently of changes in body mass^33^.

FGF21 increases browning of WAT and has been shown to enhance the expression of *Cidea*, *Ucp1* and *Pgc1α* after just 72 h of treatment^31^. FGF21 also enhances the lipid cycling process, increasing gene expression of *Scd1* and *Acc* ^35^ so could explain the effects that MR and LR had in WAT. A recent study demonstrated a dose-response effect between LR and serum FGF21; as leucine content in the diet decreased, the levels of serum FGF21 rose, which paralleled the increase in *UCP1* mRNA expression in WAT^38^. Although UCP1 was not measured in this study, this relationship between UCP1 and FGF21 corresponds to the relation present here between FGF21 and body mass loss, as UCP1 is required for MR to increase energy expenditure and, therefore, reduce body mass^16^.

In terms of insulin signalling, LR produced no measurable effect compared to control diet on insulin sensitivity in epididymal WAT. MR produced only a small effect in response to insulin stimulation, which led to increased activiation of mTOR in comparison to control diet, but no effect on activation of Akt, which has been shown previously in epididymal WAT by MR diet^28^. The lack of effect on Akt may have been a result of the low numbers used for the immunoblotting. *In vivo* MR increases levels of phosphorylation of Akt in WAT, whereas in 3T3 L1 adipocytes restricting methionine had no effect on Akt^17^. However, FGF21 treatment of 3T3 L1 adipocytes increased Akt phosphorylation, which led to the attribution of FGF21 for the effects of MR on improving WAT insulin sensitivity. This led to the conclusion that MR indirectly improves WAT insulin sensitivity, via circulating FGF21 released from the liver acting directly on WAT^17^. For LR, it is plausible that the increase in FGF21 was not sufficient enough to have an effect on WAT insulin sensitivity.

In the liver, MR and LR produced a similar response to lower activation of S6 under basal fasting conditions, but to increase levels of phosphorylation of mTOR under insulin stimulated conditions. MR produced a stronger effect to increase mTOR phosphorylation than LR, suggesting that both diets induce enhanced hepatic insulin sensitivity, but with a stronger effect of the MR diet. The variation in effects between MR and LR could be due to the distinct mechanisms of action of the diets. It was mentioned earlier that MR has methionine-specific effects to increase stimulation of Akt, through lower PTEN activation in response to insulin^17^, which would lead to increased downstream signalling, including stimulation of mTOR. Similarly, LR may have leucine-specific effects, which have been suggested by studies on leucine deprivation^24^. One study reported that that under fasting conditions, increased activation of GCN2 reduced activation of mTOR, leading to less inhibition of IRS1^24^. This would explain the lower levels of S6 phosphorylation, a protein directly activated by mTOR^24^. Under opposing conditions of insulin stimulation, LR induced significantly increased activation of mTOR, further adding to evidence for increased hepatic insulin sensitivity, in line with the effects of leucine deprivation^24^. These subtle distinctions in size of the effect on hepatic insulin sensitivity between MR and LR could play a role in the disparity of the magnitude of the effect on whole body glucose metabolism between the diets.

In conclusion, this study suggests that 80% LR is an effective dietary regime for improving metabolic health; however, 80% MR has stronger beneficial metabolic effects on body mass reduction and improvement in glucose homeostasis than 80% LR. Pathways unique to a specific EAA may cause the discrepancy in the magnitude of ﻿the effects between MR and LR, including the mechanisms by which FGF21 is induced and Akt is activated in the liver.

## Methods

### Animals

This study was completed under UK Home Office project licence PPL 60/3951 and approved by the University of Aberdeen Ethics Review Board. 10-month-old male C57BL/6 J wild-type mice (Charles River, Edinburgh, UK) were housed in pairs. Mice were exposed to 12 h light/dark cycle at 22–24 °C and had ad libitum access to food and water. Mice were placed on a control diet containing 0.86% methionine, 1.11% leucine and 2.7% glutamic acid (Dyets, Bethlehem, PA, USA) for 2 weeks. Mice were then divided evenly into three groups of 9–10 mice, randomised by body weight and fed either control (0.86% methionine, 1.11% leucine, 2.7% glutamic acid), LR (0.86% methionine, 0.22% leucine, 3.70% glutamic acid) (Dyets, Bethlehem, PA, USA) or MR (0.17% methionine, 1.11% leucine, 3.39% glutamic acid) diet (Dyets, Bethlehem, PA, USA) for 8 weeks. The glutamic acid content of the MR and LR diets was increased to compensate for the reduced methionine or leucine content, to allow for consistent amounts of total amino acids across all diets, as done previously^1, 2, 4, 16, 17, 29, 40^. Glutamic acid was used because it makes up the highest proportion in the diet among all amino acids (Dyets, Bethlehem, PA, USA). Moreover, the majority of dietary glutamate (95%) is used for energy production in the intestine^41^; therefore, it was deemed to be the least likely of all the amino acids to have any physiological effect. After 8 weeks on diet mice were fasted for 5 h, blood glucose was measured using a glucometer (AlphaTRAK, Berkshire, UK) and then the mice received an intraperitoneal (i.p.) injection with saline (154 mmol/l NaCl) or a high dose of insulin (10 mU/g body weight) and were sacrificed 10 min-post injection. After cervical dislocation, tissues were immediately dissected and frozen in liquid nitrogen.

### Whole- body measurements and blood metabolites

Throughout the study body weight and food intake were measured weekly. Body composition (fat and lean mass) was measured after 3 weeks on diet by magnetic resonance imaging (MRI) scan (Echo MRI, Houston, TX, USA). The machine was first equilibrated with a phantom mouse. The mouse was then placed into a tube and contained with a second tube to allow for only small movements of the mouse and accurate scanning. The results of the scanning included fat mass, lean mass and water content.

Glucose tolerance was assessed after 2 days and after 5 weeks on diet by a GTT. For these measurements, mice were fasted for 5 h prior to i.p. injection with glucose (2 g/kg body weight). Tail blood glucose measurements were taken using glucometers (AlphaTRAK, Berkshire, UK) immediately before and 15, 30, 60 and 120 min after i.p. injection with glucose.

Blood was collected from tail vein blood samples after 6 weeks on diet, after a 5 h fast. Serum insulin (5 µl serum) (Crystal Chem Downers Grove, Illinois, USA) and serum FGF21 (35 µl serum) (Millipore, Darmstadt, Germany) were measured from these blood samples using ELISA kits. Serum IGF-1 (10 µl serum) (R&D Systems, Minneapolis, MN, USA) was measured from trunk blood samples collected during dissection.

### Immunoblotting

Frozen liver and epididymal WAT lysates were prepared in RIPA buffer, as described previously^28^. Proteins were separated by 4–12% SDS-PAGE and transferred to nitrocellulose membranes. Immunoblots were performed using antibodies from Cell Signaling Technology (Cell Signaling by NEB, Hitchin, UK) (unless stated otherwise) against phospho-mTOR (s2448) 2971 S; total mTOR 2972; phospho-S6 (s235/236) 4858 S; total S6 2217 S; phospho-Akt/PKB (s473) 9271 S and total Akt/PKB A2210 (Santa Cruz, Dallas, Texas, USA). Proteins were visualised with enhanced chemiluminescence and quantified using Bio-1D software (Peqlab, Sarisbury Green, UK).

### Gene expression in liver and epididymal WAT

Total RNA was isolated from frozen liver and epididymal WAT using peqGOLD TriFast (Peqlab, Sarisbury Green, UK). For epididymal WAT, homogenates were centrifuged and the lipid layer was removed before phase separation. cDNA was synthesised from 1ug RNA using bioscript cDNA synthesis kit (Bioline, London, UK). Per sample the mastermix contained 0.5 μl Oligo (dT)_18_, 0.5 μl Random Hexamer, 1 μl dNTP (10 mM) mix, 4 μl 5 x RT buffer, 1 μl Ribosafe RNase Inhibitor (10 u/μl), 1 μl Tetro Reverse Transcriptase (200 u/μl), and the required volume of DEPC-treated water to make each sample up to 20 μl. Target genes were amplified by quantitative PCR (qPCR) on the LightCycler-480 (Roche, Burgess Hill, UK), using cDNA (2 µl), nuclease-free water (2.5 μl), combined forward and reverse gene-specific primers (0.5 μl) and GoTaq qPCR master mix (5 μl) (Promega, Southampton, UK). Relative mRNA levels were calculated using the Pfaffl method^42^ and normalized to a geometric mean of three reference genes; *Beta-actin*, *Glyceraldehyde 3-phosphate dehydrogenase (GAPDH)* and *Ywhaz* for the liver, and *Nono*, *Ywhaz* and *Beta-actin* for epididymal WAT.

### Statistical analysis

Data are expressed as mean ± SEM. Statistical analyses were performed using repeated measures two-way ANOVA or by two-tailed Student’s t-test, as appropriate. The ANOVA or t-tests were performed separately for each of the three comparisons (MR vs. LR, MR vs. CON, LR vs. CON). GraphPad Prism 5 software (GraphPad Software, Inc., San Diego, California, USA) was used for analyses. P-values < 0.05 were considered significant.

## Electronic supplementary material

Supplementary Info

## References

[1] Hasek BE (2010). Dietary methionine restriction enhances metabolic flexibility and increases uncoupled respiration in both fed and fasted states. Am. J. Physiol. Regul. Integr. Comp. Physiol..

[2] Plaisance EP (2010). Role of beta-adrenergic receptors in the hyperphagic and hypermetabolic responses to dietary methionine restriction. Am. J. Physiol. Regul. Integr. Comp. Physiol..

[3] Ables GP, Perrone CE, Orentreich D, Orentreich N (2012). Methionine-restricted C57BL/6J mice are resistant to diet-induced obesity and insulin resistance but have low bone density. PLoS One.

[4] Cousin B (1992). Occurrence of brown adipocytes in rat white adipose tissue: molecular and morphological characterization. J. Cell. Sci..

[5] Hasek BE (2013). Remodeling the integration of lipid metabolism between liver and adipose tissue by dietary methionine restriction in rats. Diabetes.

[6] Perrone CE, Mattocks DA, Jarvis-Morar M, Plummer JD, Orentreich N (2010). Methionine restriction effects on mitochondrial biogenesis and aerobic capacity in white adipose tissue, liver, and skeletal muscle of F344 rats. Metabolism.

[7] Perrone CE (2008). Methionine restriction effects on 11 -HSD1 activity and lipogenic/lipolytic balance in F344 rat adipose tissue. J. Lipid Res..

[8] Anthony TG, Morrison CD, Gettys TW (2013). Remodeling of lipid metabolism by dietary restriction of essential amino acids. Diabetes.

[9] Mottillo EP (2014). Coupling of lipolysis and de novo lipogenesis in brown, beige, and white adipose tissues during chronic beta3-adrenergic receptor activation. J. Lipid Res..

[10] Masoro EJ (1963). Role of lipogenesis in nonshivering thermogenesis. Fed. Proc..

[11] Dulloo AG, Gubler M, Montani JP, Seydoux J, Solinas G (2004). Substrate cycling between de novo lipogenesis and lipid oxidation: a thermogenic mechanism against skeletal muscle lipotoxicity and glucolipotoxicity. Int. J. Obes. Relat. Metab. Disord..

[12] Yu XX, Lewin DA, Forrest W, Adams SH (2002). Cold elicits the simultaneous induction of fatty acid synthesis and beta-oxidation in murine brown adipose tissue: prediction from differential gene expression and confirmation *in vivo*. FASEB J..

[13] Richie JP (1994). Methionine restriction increases blood glutathione and longevity in F344 rats. FASEB J..

[14] Miller RA (2005). Methionine-deficient diet extends mouse lifespan, slows immune and lens aging, alters glucose, T4, IGF-I and insulin levels, and increases hepatocyte MIF levels and stress resistance. Aging Cell..

[15] Wanders D, Ghosh S, Stone KP, Van NT, Gettys TW (2014). Transcriptional impact of dietary methionine restriction on systemic inflammation: relevance to biomarkers of metabolic disease during aging. Biofactors.

[16] Wanders D (2015). UCP1 is an essential mediator of the effects of methionine restriction on energy balance but not insulin sensitivity. FASEB J..

[17] Stone KP, Wanders D, Orgeron M, Cortez CC, Gettys TW (2014). Mechanisms of increased *in vivo* insulin sensitivity by dietary methionine restriction in mice. Diabetes.

[18] Malloy VL (2013). Methionine restriction prevents the progression of hepatic steatosis in leptin-deficient obese mice. Metabolism.

[19] Malloy VL (2006). Methionine restriction decreases visceral fat mass and preserves insulin action in aging male Fischer 344 rats independent of energy restriction. Aging Cell..

[20] De Marte ML, Enesco HE (1986). Influence of low tryptophan diet on survival and organ growth in mice. Mech. Ageing Dev..

[21] Stoltzner G (1977). Effects of life-long dietary protein restriction on mortality, growth, organ weights, blood counts, liver aldolase and kidney catalase in Balb/C mice. Growth.

[22] Cheng Y (2010). Leucine deprivation decreases fat mass by stimulation of lipolysis in white adipose tissue and upregulation of uncoupling protein 1 (UCP1) in brown adipose tissue. Diabetes.

[23] Cheng Y (2011). Leucine deprivation stimulates fat loss via increasing CRH expression in the hypothalamus and activating the sympathetic nervous system. Mol. Endocrinol..

[24] Xiao F (2011). Leucine deprivation increases hepatic insulin sensitivity via GCN2/mTOR/S6K1 and AMPK pathways. Diabetes.

[25] De Sousa-Coelho AL, Marrero PF, Haro D (2012). Activating transcription factor 4-dependent induction of FGF21 during amino acid deprivation. Biochem. J..

[26] De Sousa-Coelho AL (2013). FGF21 mediates the lipid metabolism response to amino acid starvation. J. Lipid Res..

[27] Wanders D (2016). Role of GCN2-Independent Signaling Through a Noncanonical PERK/NRF2 Pathway in the Physiological Responses to Dietary Methionine Restriction. Diabetes.

[28] Lees EK (2014). Methionine restriction restores a younger metabolic phenotype in adult mice with alterations in fibroblast growth factor 21. Aging Cell..

[29] Badman MK (2007). Hepatic fibroblast growth factor 21 is regulated by PPARalpha and is a key mediator of hepatic lipid metabolism in ketotic states. Cell. Metab..

[30] Inagaki T (2007). Endocrine regulation of the fasting response by PPARalpha-mediated induction of fibroblast growth factor 21. Cell. Metab..

[31] Fisher FM (2012). FGF21 regulates PGC-1alpha and browning of white adipose tissues in adaptive thermogenesis. Genes Dev..

[32] Kharitonenkov A (2008). FGF-21/FGF-21 receptor interaction and activation is determined by betaKlotho. J. Cell. Physiol..

[33] Xu J (2009). Fibroblast growth factor 21 reverses hepatic steatosis, increases energy expenditure, and improves insulin sensitivity in diet-induced obese mice. Diabetes.

[34] Badman MK, Koester A, Flier JS, Kharitonenkov A, Maratos-Flier E (2009). Fibroblast growth factor 21-deficient mice demonstrate impaired adaptation to ketosis. Endocrinology.

[35] Coskun T (2008). Fibroblast growth factor 21 corrects obesity in mice. Endocrinology.

[36] Zhang Y (2011). The link between fibroblast growth factor 21 and sterol regulatory element binding protein 1c during lipogenesis in hepatocytes. Mol. Cell. Endocrinol..

[37] Potthoff MJ (2009). FGF21 induces PGC-1alpha and regulates carbohydrate and fatty acid metabolism during the adaptive starvation response. Proc. Natl. Acad. Sci. USA.

[38] Wanders D (2015). Metabolic responses to dietary leucine restriction involve remodeling of adipose tissue and enhanced hepatic insulin signaling. Biofactors.

[39] Cullinan SB (2003). Nrf2 is a direct PERK substrate and effector of PERK-dependent cell survival. Mol. Cell. Biol..

[40] Nguyen T, Nioi P, Pickett CB (2009). The Nrf2-antioxidant response element signaling pathway and its activation by oxidative stress. J. Biol. Chem..

[41] Reeds PJ, Burrin DG, Stoll B, Jahoor F (2000). Intestinal glutamate metabolism. J Nutr..

[42] Pfaffl MW (2001). A new mathematical model for relative quantification in real-time RT-PCR. Nucleic Acids Res..

