# Supplementary material for: Direct comparison of methionine restriction with leucine restriction on the metabolic health of C57BL/6J mice
Source: Sci Rep. 2017 Aug 30;7:9977. doi: 10.1038/s41598-017-10381-3 (PMC5577041; doi:10.1038/s41598-017-10381-3)
Supplement: Supplementary file 1 — Supplementary Info [file 41598_2017_10381_MOESM1_ESM.pdf]

Direct comparison of methionine restriction with leucine restriction on the metabolic health of C57BL/6J mice.

Emma K. Lees[1,2], Ruth Banks[1], Chelsea Cook[1], Sophie Hill[1], Nicola Morrice[1], Louise Grant[1], Nimesh Mody[1] & Mirela Delibegovic[1]*

[1]Institute of Medical Sciences, School of Medical Sciences, University of Aberdeen, Aberdeen, UK

[2] School of Health Sciences, Liverpool Hope University, Liverpool, UK

[*]Corresponding author:

Professor Mirela Delibegović

Address: Institute of Medical Sciences, School of Medical Sciences, University of Aberdeen, Foresterhill, Aberdeen, AB25 2ZD, UK.

Tel: +44 1224 437587

Fax: +44 1224 437465

Email: m.delibegovic@abdn.ac.uk

Supplementary western blot data

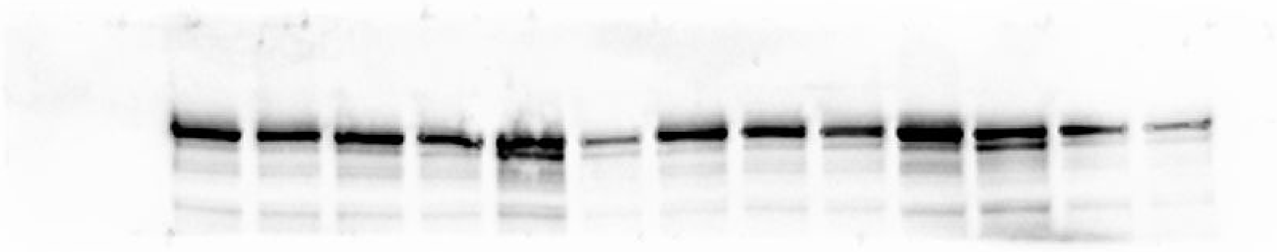

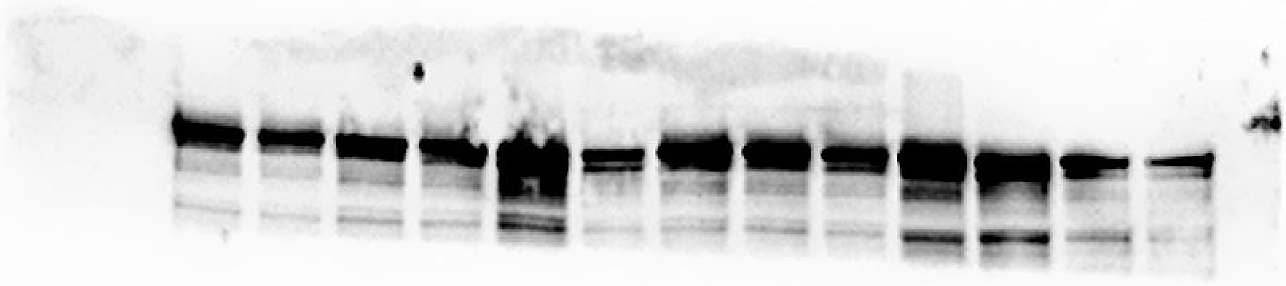

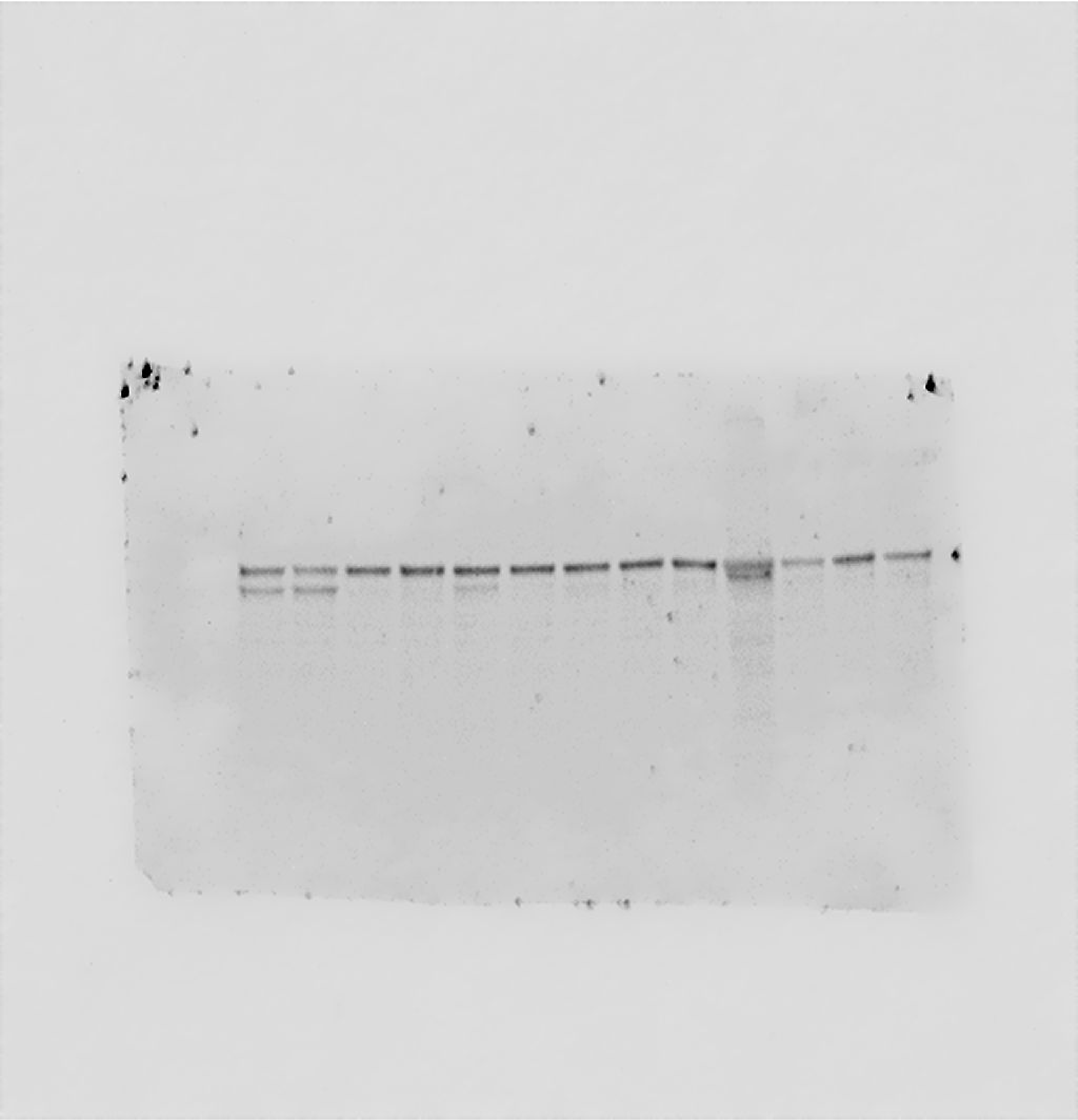

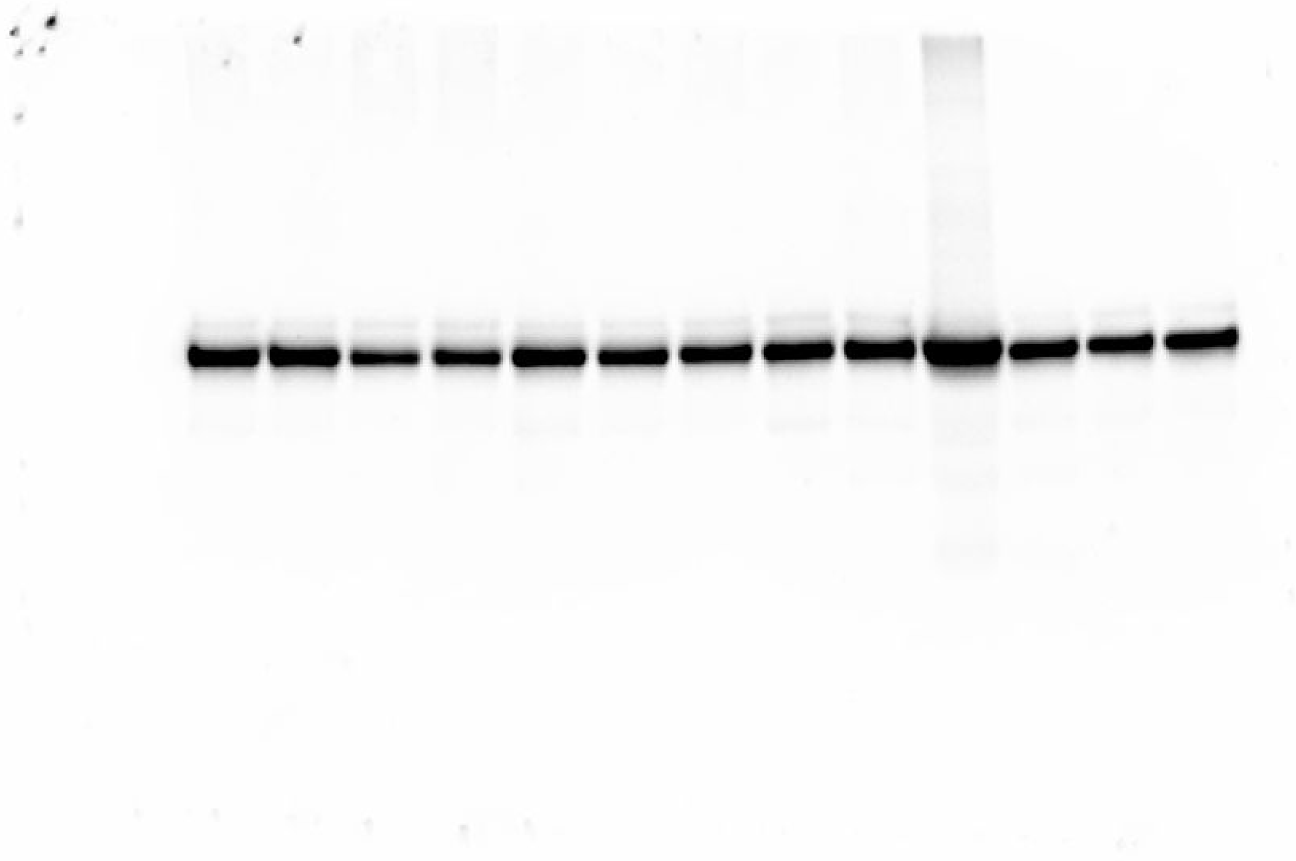

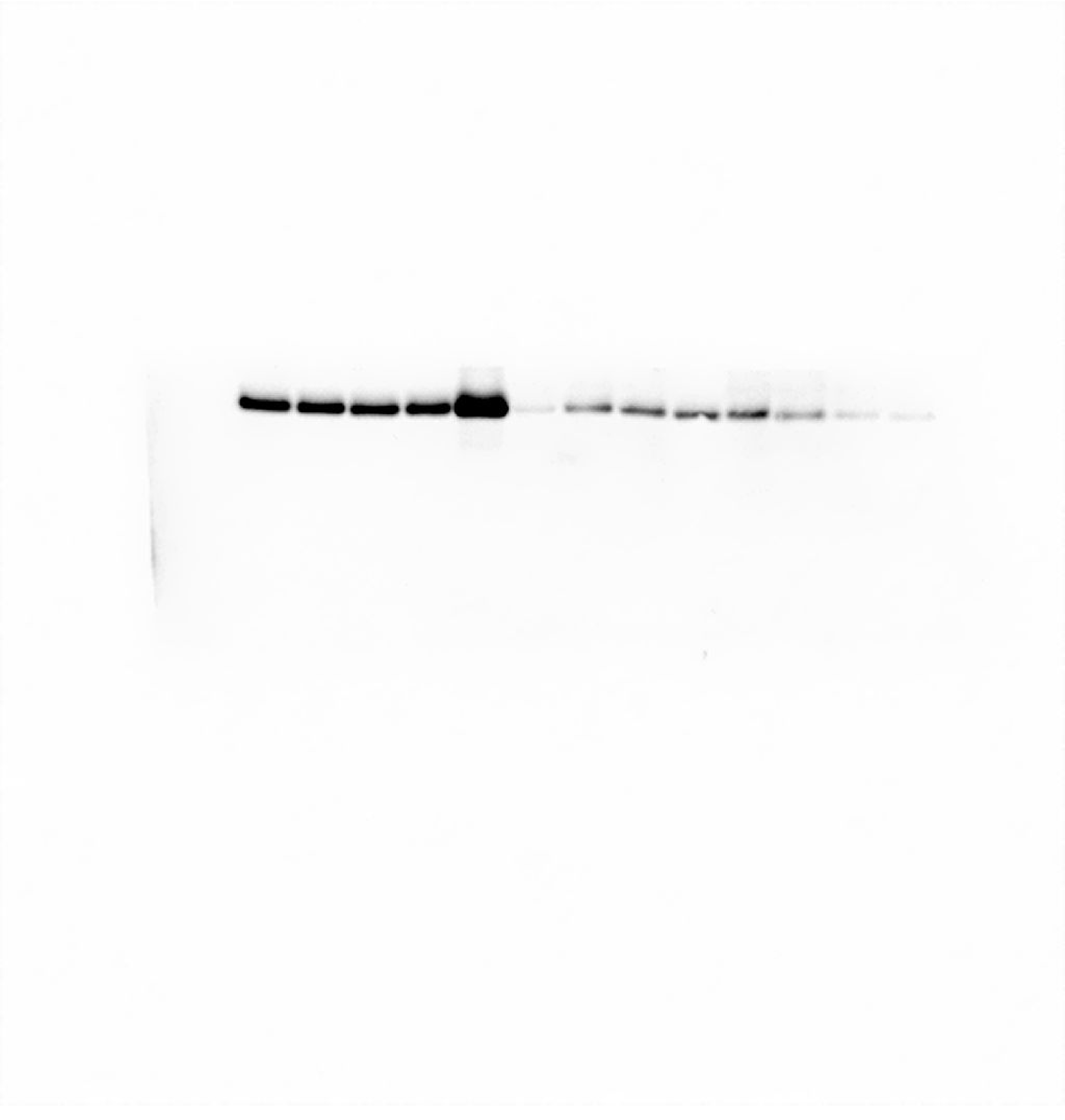

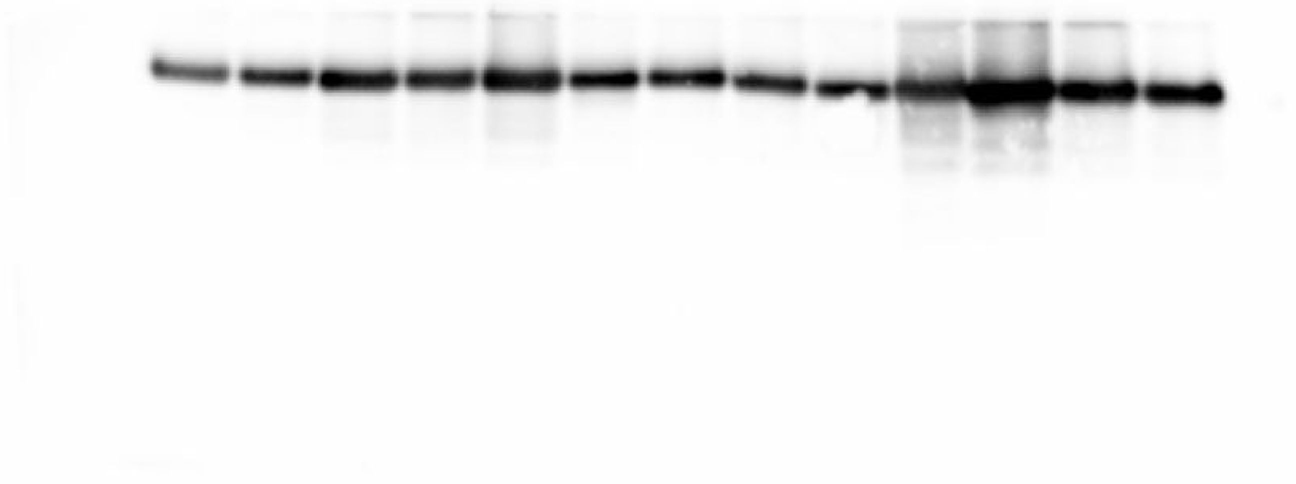

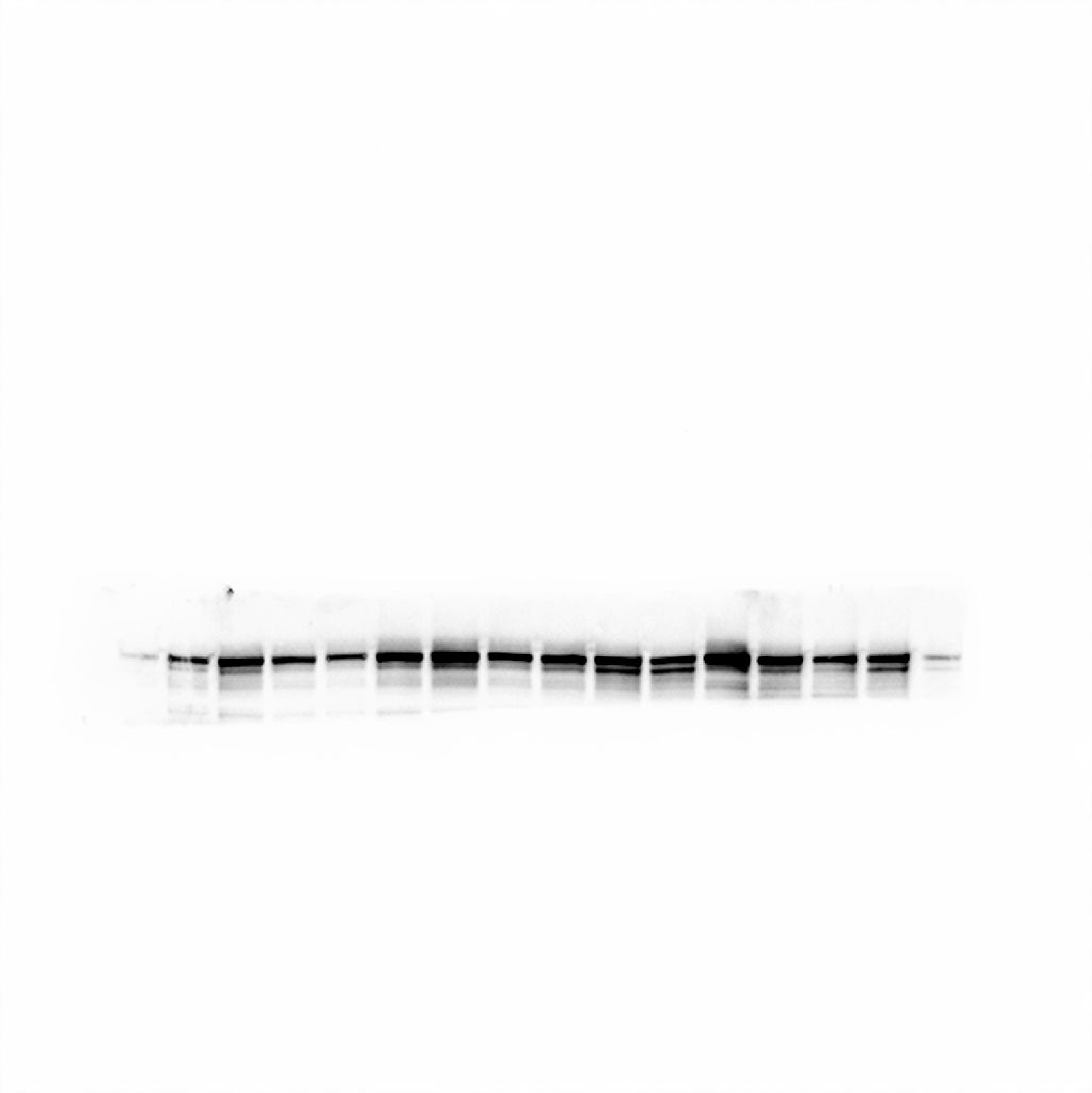

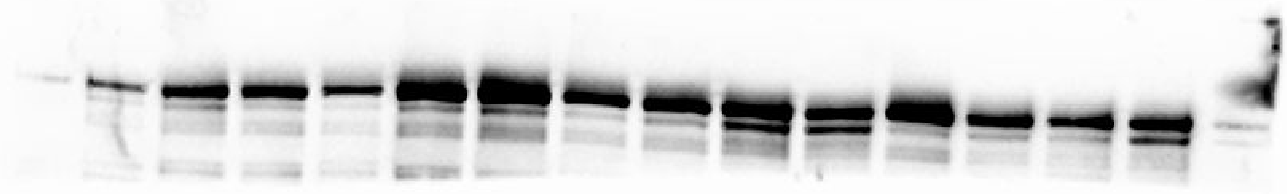

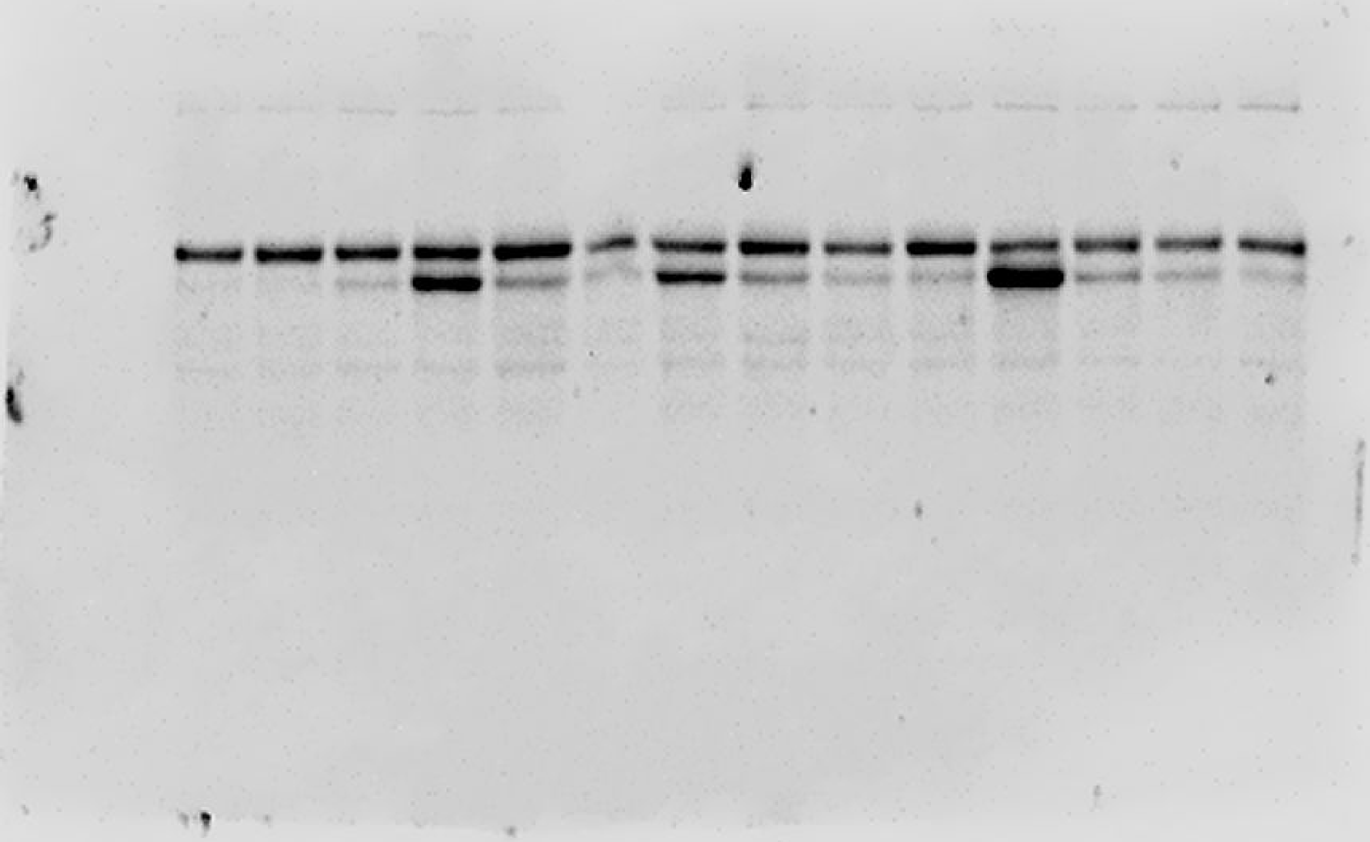

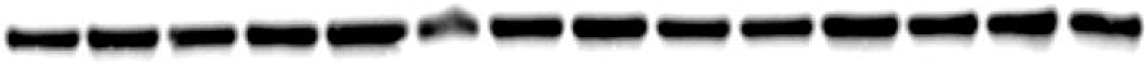

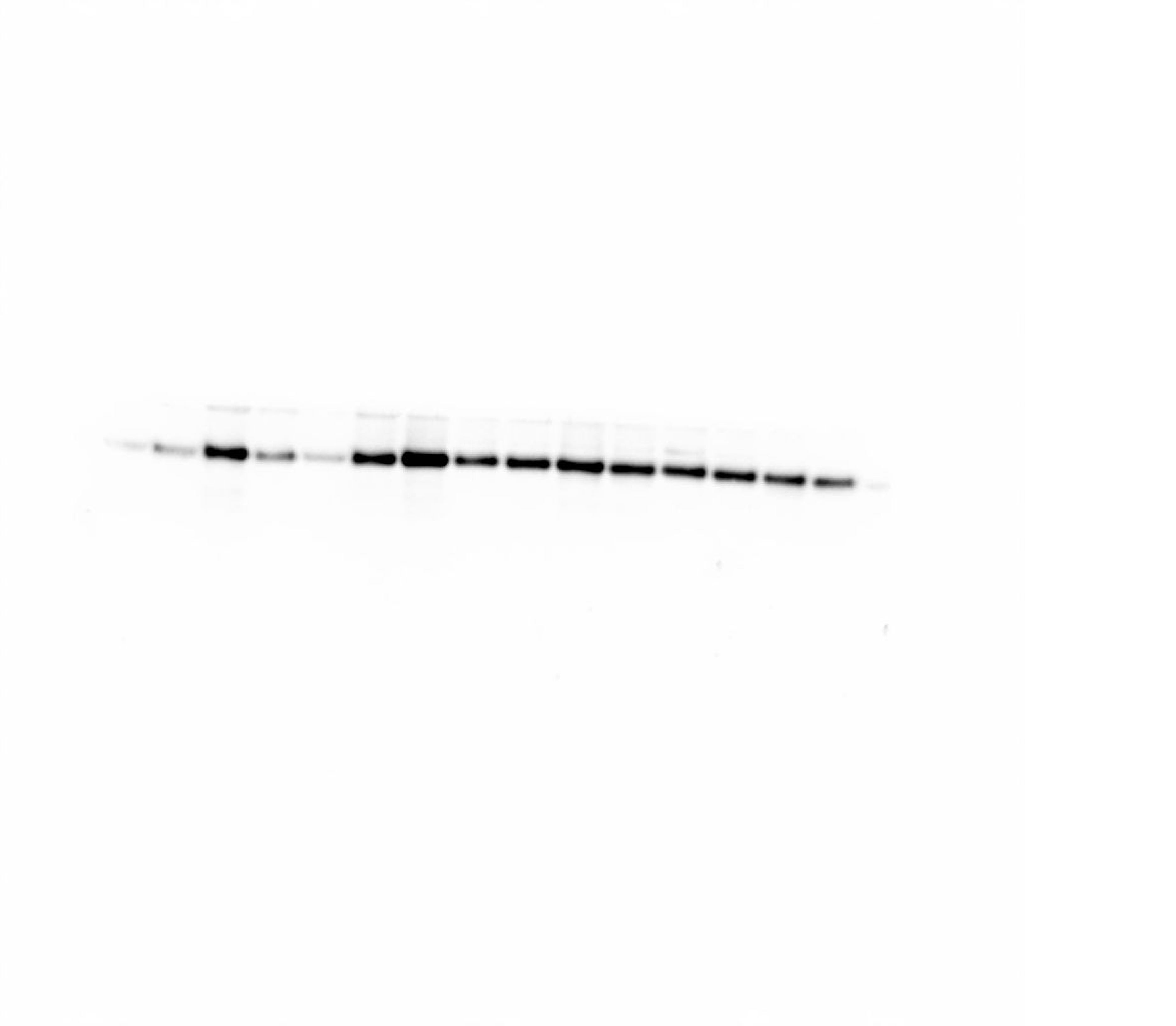

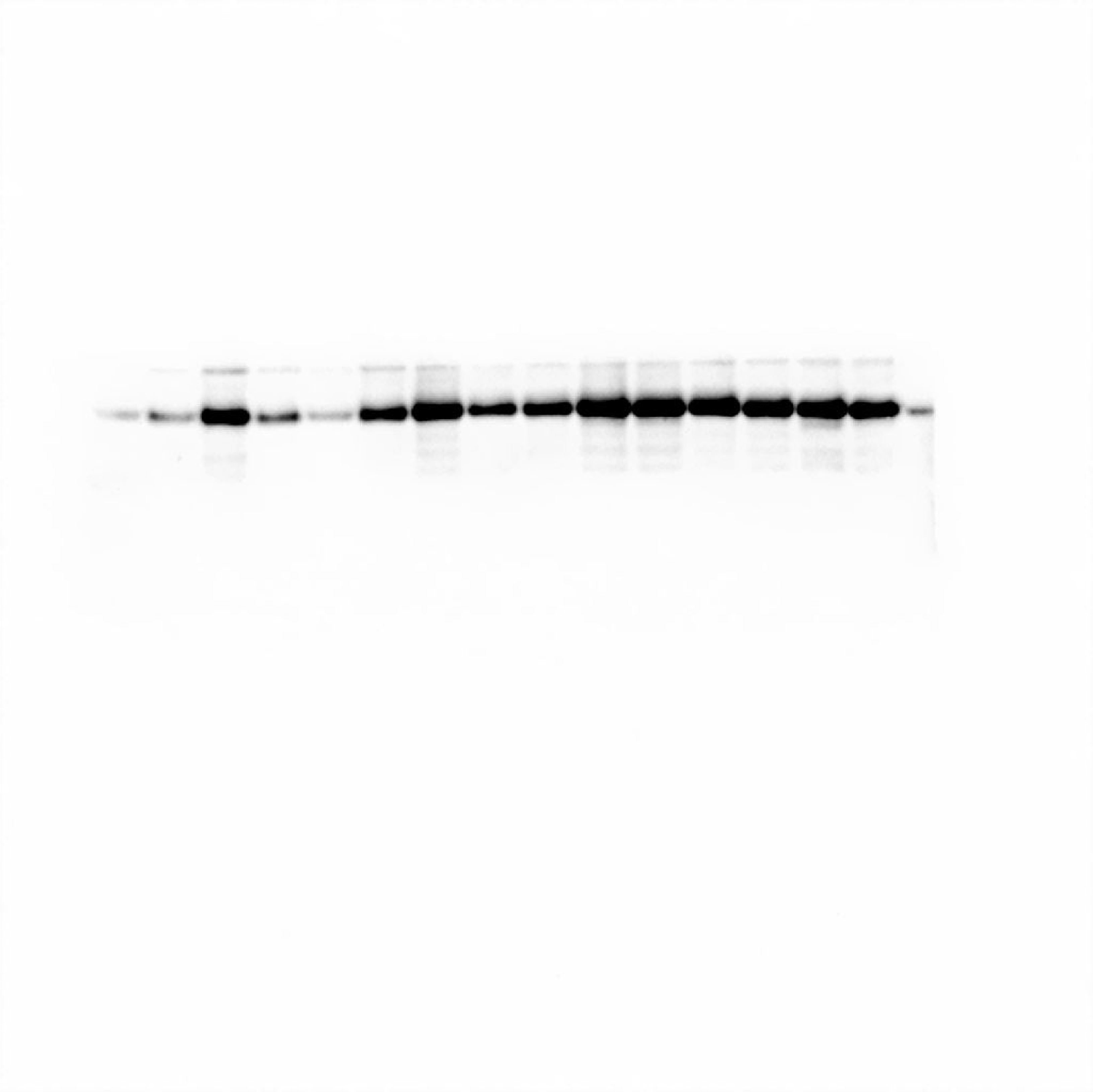

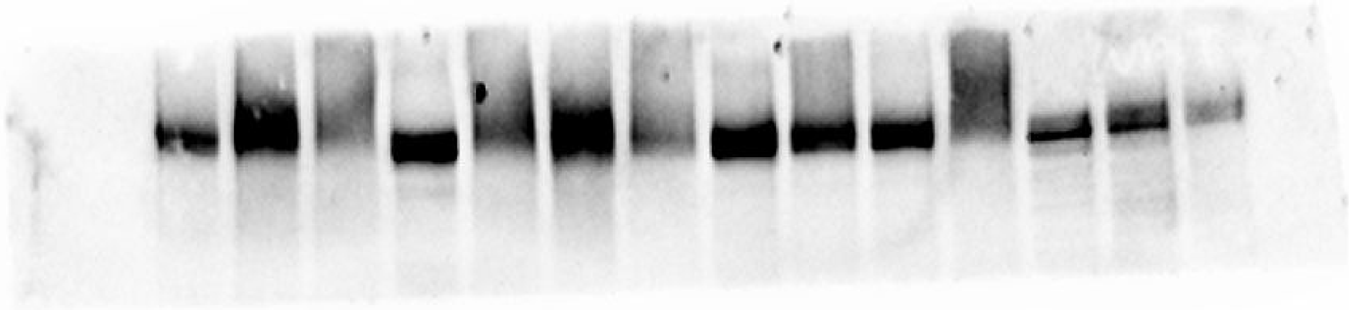

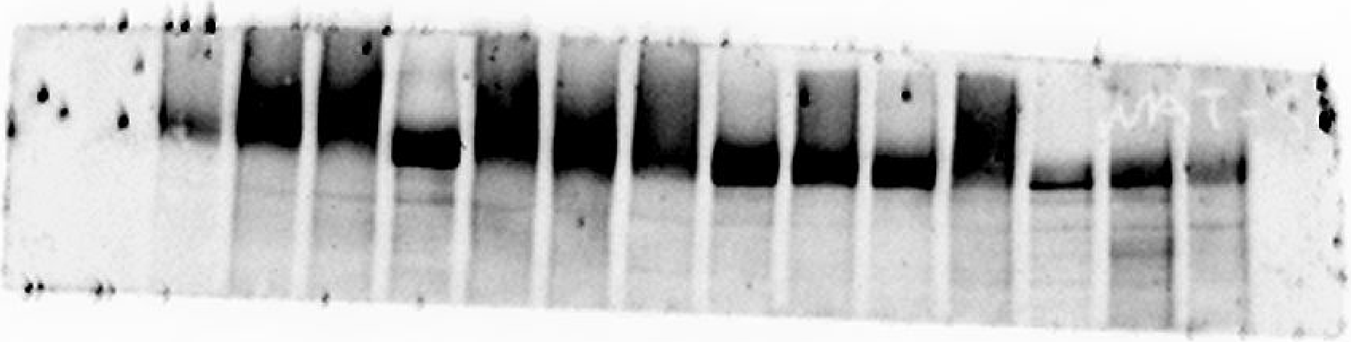

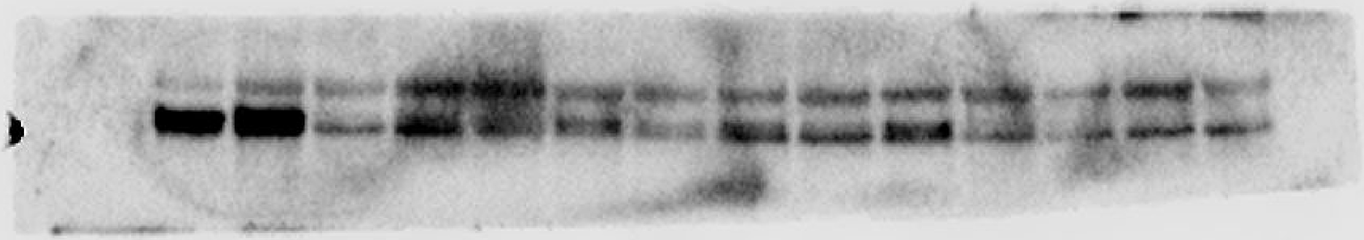

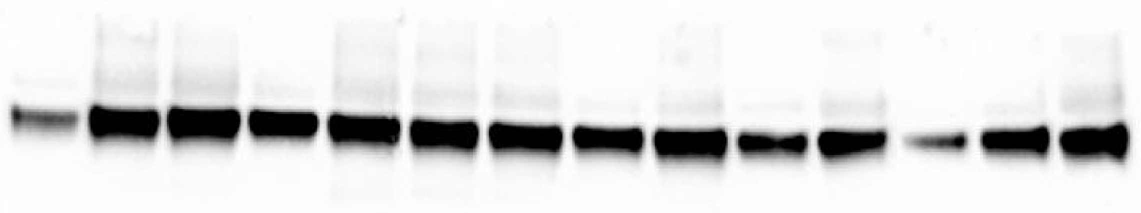

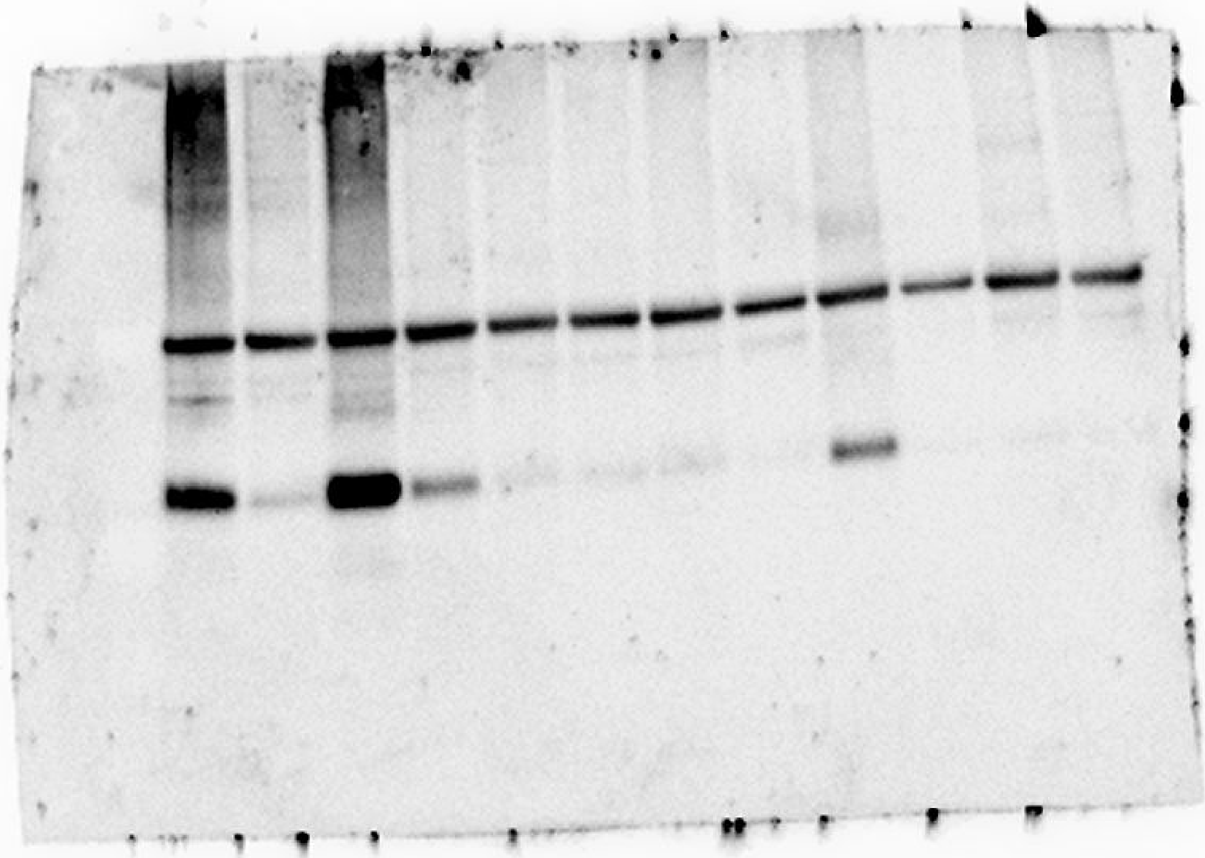

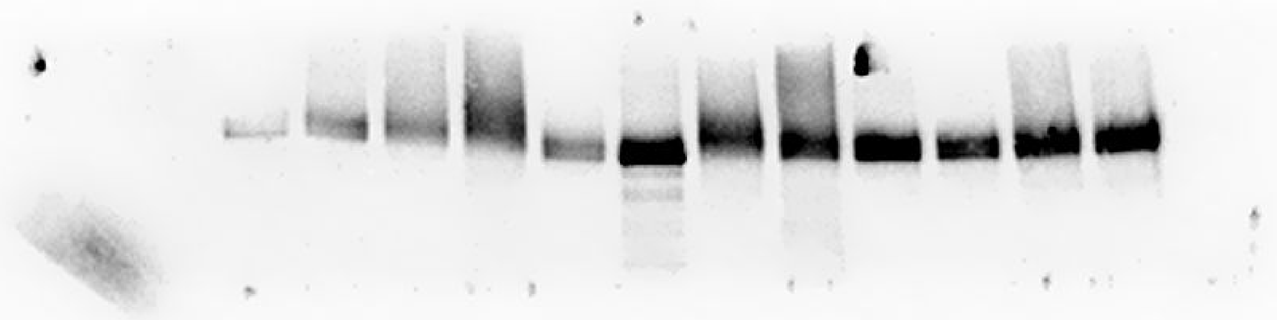

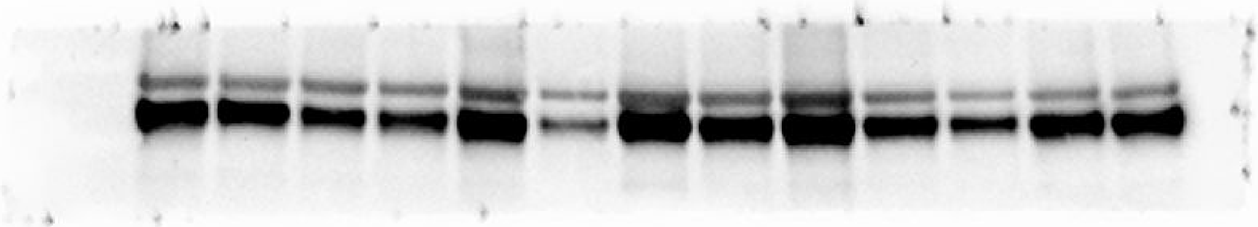

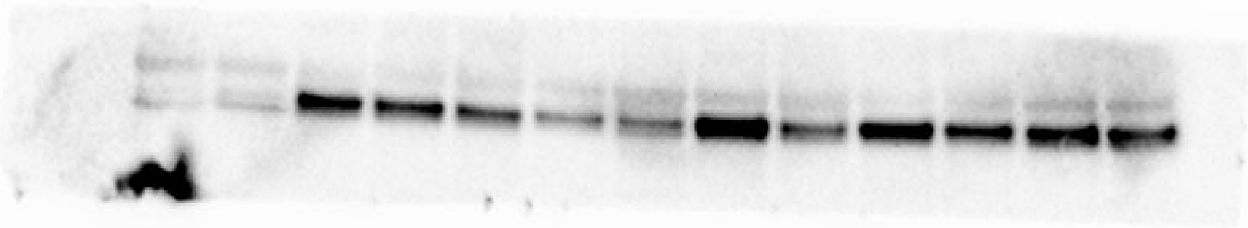

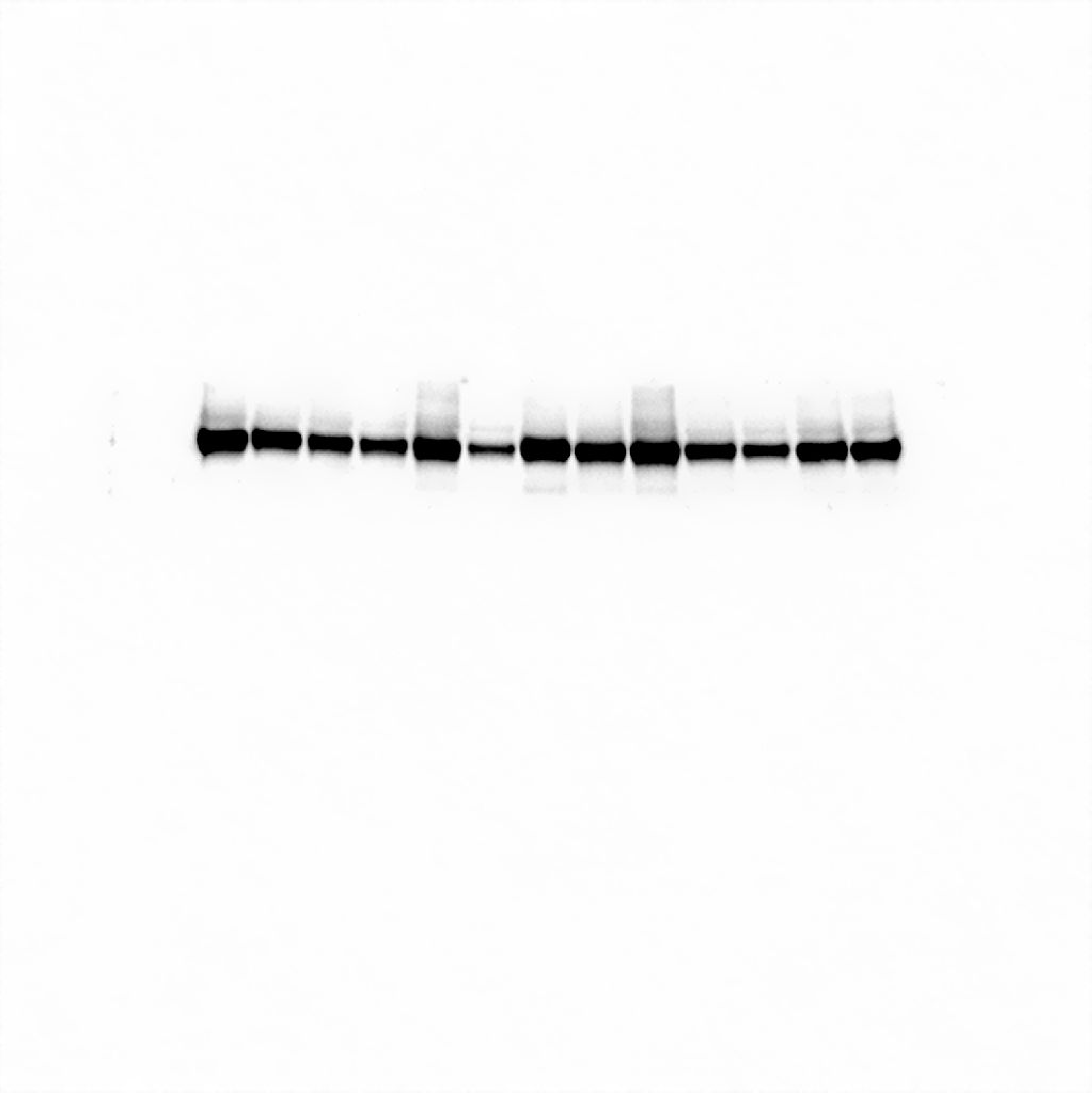

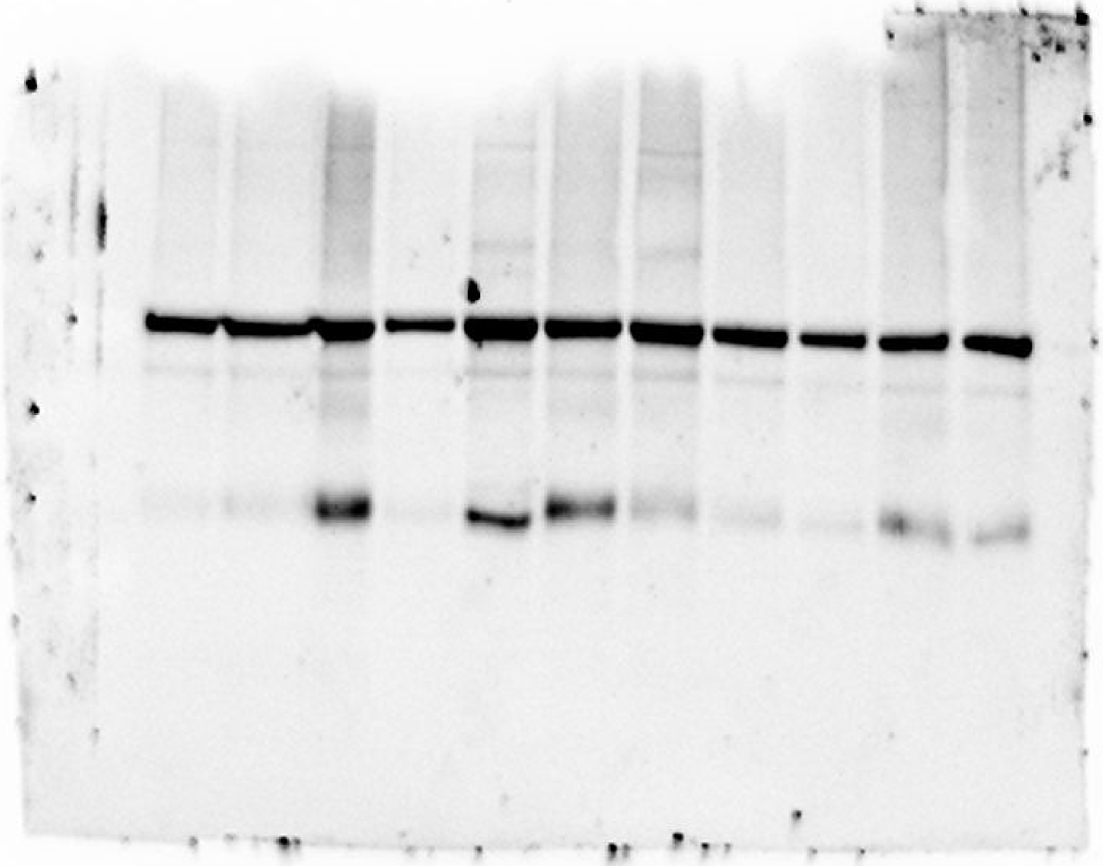

Figure legend

First two bands on every blot are control samples and were taken off for final figures as were not run as part of the analysis.

Hepatic insulin signalling in mice on MR (*n* = 4), LR (*n* = 4) and control (*n* = 3-4) diet. Data were analysed as fold change relative to control-fed mice. Insulin signalling was assessed by administering either saline (154 mmol/l NaCl) or high dose of insulin (10 mU/g body weight) via i.p. injection after a 5 h fast. Levels of phosphorylated and total mTOR, Akt and S6 were measured my immunoblotting. Immunoblots were normalised to total protein for saline condition and insulin-stimulated condition. Significance was calculated by two-tailed Student's t-test. [a,b]Means with letters that differ denote significance at level of $P < 0.05$. Data are represented as mean ± SEM. White bars, control-fed mice; grey bars, LR-fed mice; black bars, MR-fed mice.

1 – p-mTOR saline

2 – Total mTOR saline

3 – pAkt saline

4 – Total Akt saline

5 – pS6 saline

6 – Total S6 saline

7 – p-mTOR insulin

8 – Total mTOR insulin

9 – pAkt insulin

10 – Total Akt insulin

11 – pS6 insulin

12 – Total S6 insulin

Epididymal WAT insulin signalling in mice on MR (*n* = 4), LR (*n* = 4) and control (*n* = 3-4) diet. Insulin signalling was assessed by administering either saline (154 mmol/l NaCl) or high dose of insulin (10 mU/g body weight) via i.p. injection after a 5 h fast. Levels of phosphorylated and total mTOR, Akt and S6 were measured my immunoblotting. Immunoblots were normalised to total protein for saline condition and insulin-stimulated condition. Data were analysed as fold change relative to control-fed mice. Significance was calculated by two-tailed Student's t-test. [a,b]Means with letters that differ denote significance at level of $P < 0.05$. Data are represented as mean ± SEM. White bars, control-fed mice; grey bars, LR-fed mice; black bars, MR-fed mice.

13 – p-mTOR saline

14 – Total mTOR saline

15 – pAkt saline

16 – Total Akt saline

17 – pS6 (lower band) and Total S6 (higher band) saline

18 – p-mTOR insulin

19 – Total mTOR insulin

20 – pAkt insulin

21 – Total Akt insulin

22 – pS6 (lower band) and Total S6 (higher band) insulin
